# Quality of life and factors associated among caregivers of adolescent and young adult Ebola survivors in Democratic Republic of the Congo, a cross-sectional study

**DOI:** 10.1186/s12889-023-17222-1

**Published:** 2023-11-29

**Authors:** Kisughu Munyumu, Charles Wafula, Vincent Were, Françoise Katungu, Ndemo Mbasa, Margaret Kaseje

**Affiliations:** 1 Department of Paediatrics, School of Medicine, Goma University, College of Health, PO Box 03, Goma, Democratic Republic of the Congo; 2 Department of Community Health and Development, School of Health Science, Great Lake of Kisumu, P.O. Box 2224, Kisumu, Kenya; 3 Department of Community Health and Development, Université Libre Des Pays Des Grands Lacs, P.O. Box 36, Goma, Democratic Republic of the Congo

**Keywords:** Quality of life, Ebola survivors, Adolescent, Young adult, Caregivers, DRC

## Abstract

**Background:**

Ebola virus disease is a medical condition whose consequent effects on quality of life of patients. In the history of infectious diseases, there have been pathologies that have had significant repercussions for caregivers, healthcare providers and the community.

**Objectives:**

This study investigate determinants of quality of life among caregivers of adolescent and young adult Ebola survivors in Democratic Republic of the Congo.

**Methods:**

This was a cross sectional study. The study sites were the two health districts of Beni and Katwa, in North-Kivu province in the Eastern part of Democratic Republic of the Congo. The study period was from April to August 2022. Participants of the study were caregivers of adolescents and young adult Ebola virus survivors. Simple random sampling technique was used to select the 68 study participants. A questionnaire was administered. Data was collected using pretested questionnaire of WHO quality of life Bref (WHOQOL-BREF) and CommCare by Dimagi.Inc. lastest Version 2.52.1 and  a sum of score of 78 or higher indicated a high level of life quality. To determine the quality of life of caregivers of adolescents and young adult EVD survivors, descriptive analysis was used. The Pearson correlation coefficient was utilized to check whether the predictor variables are multicollinear. The regression analysis produced the crude odds ratio (COR), adjusted odds ratio (aOR), 95% confidence interval (CI), and *p*-value. Statistical significance was defined as a *p*-value 0.05. The final multivariate model contained variables that were significant in the bivariate analysis. Prior to data collection, a research permit from National Ethical Committee of Research in Democratic Republic of the Congo was obtained. Written informed consents from literate or illiterate caregivers of adolescent and young adult Ebola survivors were obtained. Throughout the study, participants' privacy and confidentiality were respected.

**Results:**

A total of 68 care givers participated in the study, with a majority 54/68(79.41%) having poor quality of life. Men were 3.17 times more likely to record good quality of life than women (*p* = 0.02); OR:(95% CI), 3.17: (1.2 – 8.36), With regards to place of residence, caregivers who lived in town were less likely to have good quality of life compared to those in rural (*p* = 0.01); OR: (95%CI), 0.25: (0.09 – 0.72).

**Conclusion:**

The quality of life of caregivers of adolescent and young adult Ebola survivors in Democratic Republic of the Congo is poor. To be woman caregiver and to live in town are determinants associated with poor quality of life among caregivers of adolescent and young adult Ebola survivors.

## Background

In Democratic Republic of the Congo, Ebola Virus disease was first encountered in 1976. Since then, there were more than 14 outbreaks in the region, with the frequency of occurrence increasing drastically over time [1]. This has been compounded by economic, sociocultural factors, political instability, and slow disease response in the region as well as globally [2]. Despite its high morbidity and mortality, there has been a substantial number of survivors who upon discharge from treatment centres, suffer short term or long-term physical, emotional, and social sequelae.

For this reason, the need for caregivers to manage the disease and support these survivors have intensified [3, 4]. Care giving is demanding and overwhelming, and can take a toll on one’s emotional, financial and psychological wellbeing specially in cases of chronic health condition and infectious diseases [5]. In most cases, caregivers assume the role with little or no information about the disease or caregiving services. These predispositions, together with other factors profoundly impact the quality of life of the caregivers who play an integral role in the management of the disease, patients and the survivors’ recovery [6, 7].

Studies have been conducted on the quality of life of caregivers of patients with chronic illnesses [3, 8]. Additionally, there is a gradually growing body of research on Ebola Virus and its effects on survivors particularly with regards to their health and their quality of life post recovery [6, 9, 10]. However, there is limited research and knowledge on the challenges, experiences and quality of life of the caregivers who are so much involved in these survivors’ lives. The purpose of this research is to examine the quality of life and associated factors among caregivers of Adolescent and young adult Ebola survivors in DRC Congo.

## Methods

### Study design

This was a cross-sectional study.

### Setting

The study was conducted in the health districts of Beni and Katwa in North Kivu, Democratic Republic of the Congo (DRC). North Kivu is one of the provinces in Eastern part of DRC and has a population of about 1.9 million. It borders Lake Kivu.

### Study period

The study was carried out during a 4 months’ period from April to August 2022.

### Study participants

Participants of the study were caregivers of adolescents and young adult Ebola virus survivors. Random sampling technique was used to select the 68 study participants.

Caregivers of adolescents and young adult Ebola virus survivors were enrolled when they stayed for 4 months with adolescents or young adult Ebola virus survivors in the study setting.

Caregivers had a thorough history and a complete physical examination undertaken to capture their social demographics, co morbidities as well as the bio-medical characteristics, using the designed questionnaire for data collection. Further information regarding other chronic illnesses (asthma, renal, hepatic, cardiac impairment and known cases of symptomatic HIV/AIDS) as well as chronic medication was also obtained through reviewing the caregiver’s medical records. Severely ill caregivers with chronic health problems and who were unable to provide adequate information were excluded to the study.

The provision of written informed consent was required for all study participants before enrolment into the study.

### Sample size calculations

A total sample size of 68 participants was estimated using the Kish Leslie (1965) formula for finite populations, based on determinants of quality of life among adolescent and young adult Ebola survivors in Democratic Republic [10]. The formula for the sample size of surveys i.e. the Kish Leslie (1965) formula below was used (adjusted for available population): sample size = n/1 + n/N, where n = z^2^ x p (1-p)/e^2^.

### Study procedures

Data was collected using CommCare by Dimagi.Inc. Version 2.52.1. Sociodemographic, community and medical variables were collected. Socio-demographic variables included; age (25–55 and > 55), sex, education level (None/Primary, Secondary, higher), marital status (married, single), residence (rural, urban), religion (protestant, catholic, other) and socio-economic status. The medical variables were muscle pain, chest pain, fatigue, sleep problems. Community variables wee social support, stigma, experienced stress. Data was collected using the interviewer administered pretested questionnaires of WHO quality of life BREF (WHOQOL-BREF). A total score < 78 was regarded as poo quality of life and a sum of ≥ 78 indicates good quality of life [10, 11]. A Likert scale was used to measure the quality of life ranging from 1- not at all, 2- slight, 3- moderate, 4- very, 5- complete. The assessed parameters on WHO quality of life BREF were physical health, psychological health, social health and environment health.

Data were collected by trained research assistants who interviewed the caregivers of adolescents and young adult EVD survivors.

### Statistical analysis

Data was entered using CommCare by Dimagi.Inc. lastest Version 2.52.1 and exported into STATA version 12 for analysis. Descriptive analysis was used. Categorical variables were analysed using frequencies, proportions and percentages. The regression analysis produced odds ratio (OR), 95% confidence interval (CI), and *p*-value. The final multivariate model contained variables that were significant in the bivariate analysis. The research used the assumption that each pair of outcomes had a proportional chance of being either bad or good quality of life. The factors with *P-*values less than 0.05 were considered significant.

### Ethical aspects

Prior to data collection, a research permit from National Ethical Committee of Research in Democratic Republic of Congo and Great Lake University of Kisumu-Kenya, helped to alleviate mistrust and allowed the participants to reveal much of the information required for the study. Written informed consents were obtained from literate or illiterate caregivers of adolescent and young adult Ebola survivors before enrolment into the study. Throughout the study, participants' privacy and confidentiality were respected.

## Results

Out of 68 caregivers who participated in the study, 48.5% (33/68) were females and 51.5% (35/68) were males. The mean age(± SD) of the participants was 46.68(16.17) years. A majority were between the age 25–55 years 63.2% (43/68) and > 55 years 36.8% (25/68). Catholic religion was the most dominant religion 54.41% (37/68) followed by protestants 30.88% (21/68), see Table 1.

**Table 1:** Participants’ socio-demographic determinants

Variables	Female		Male		Total	
	**n (33)**	**%**	**n (35)**	**%**	**n (68)**	**%**
**Sociodemographic determinants**						
**Age**						
25–55	23	27.27	20	42.86	43	63.2
> 55	10	30.30	15	42.86	25	36.8
**Marital status**						
Married	13	39.39	25	71.43	38	55.88
Single	20	60.61	10	28.57	30	44.12
**Residence**		0.00		0.00		
Rural	10	30.30	7	20.00	17	25
Town	23	69.70	28	80.00	51	75
**Occupation**		0.00		0.00		
Unemployed	28	84.85	16	45.71	44	64.71
Employed	5	15.15	19	54.29	24	35.29
**Education**						
Primary/none	20	60.61	20	57.14	40	58.82
Secondary or higher	13	39.39	15	42.86	28	41.18
**Religion**						
Protestant	11	33.33	10	28.57	21	30.88
Catholic	17	51.52	20	57.14	37	54.41
Other	5	15.15	5	14.29	10	14.71
**Shelter**						
Yes	28	84.85	30	85.71	58	85.29
No	5	15.15	5	14.29	10	14.71
**Household head**						
Yes	5	15.15	6	17.14	11	16.18
No	28	84.85	29	82.86	57	83.82
**Household number**						
4–7	12	36.36	23	65.71	35	51.47
> 7	21	63.64	12	34.29	33	48.53
**Monthly allowance**						
< 10 $	16	48.48	5	14.29	21	30.88
> 10 $	17	51.52	30	85.71	47	69.12

**Fig. 1:**
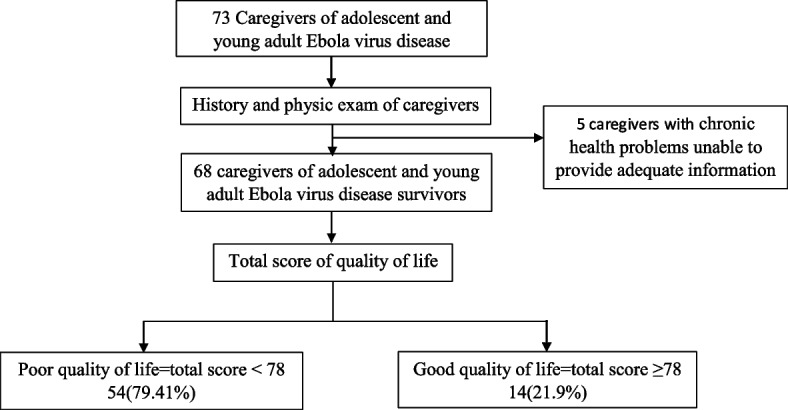
Study profile

Seventy-four percent (54/68) of study participants had poor quality of life while 20.59% (14/68) had good quality of life, see Fig. 1, Table 2.

**Table 2:** Quality of life

Quality of life status	n (68)	%
Poor	54	79.41
Good	14	20.59

A total of 68 caregivers participated in the survey, with a majority 79.41% (54/68) having poor quality of life while 20.59 % (14/68) having good quality of life, see Table 2.

In terms of medical factors, results from the table show that, approximately, 25. % (17/68) have fatigue; 23.53% (16/68) have muscle pain;17.65% (12/68) have sleep problem and 13.24% (9/68) have chest pain. With regards to community factors, almost three quarter of the caregivers who participated in the study 85.29% (58/68) had experienced stress, 70.59% (48/68) had experienced stigma and 58.82% (40/68) reported no social support, see Table 3.

**Table 3:** Participants’ medical and community determinants

Variables	Female		Male		Total	
	**n (33)**	**%**	**n (35)**	**%**	**n (68)**	**%**
**Medical determinants**						
**Muscle pain**						
Yes	12	36.36	4	11.43	16	23.53
No	21	63.64	31	88.57	52	76.47
**Chest Pain**						
Yes	9	27.27	0	0.00	9	13.24
No	24	72.73	35	100.00	59	86.76
**Fatigue**						
Yes	14	42.42	3	8.57	17	25.00
No	19	57.58	32	91.43	51	75.00
**Sleep problem**						
Yes	9	27.27	3	8.57	12	17.65
No	24	72.73	32	91.43	56	82.35
**Community factors**						
**Stigma**						
Yes	24	72.73	24	68.57	48	70.59
No	9	27.27	11	31.43	20	29.41
**Experienced Stress**						
Yes	28	84.85	30	85.71	58	85.29
No	5	15.15	5	14.29	10	14.71
**Social support**						
Yes	17	51.52	11	31.43	28	41.18
No	16	48.48	24	68.57	40	58.82

Markers of sex and occupation were determinants associated with quality of life on bivariate analysis (*p* = 0.01), see Table 4.

**Table 4:** Bivariate analysis for socio-demographic, medical and community determinants

Variables	Unadjusted OR	95% Cl	*P*-value
**Sociodemographic determinants**			
**Age**			
26–55	Ref		
> 55	1.48	0.40—5.51	0.561
**Sex**			
Female	Ref		
Male	8.09	1.64—39.70	**0.01**
**Marital status**			
Married	Ref		
Single	0.43	0.12—1.54	**0.196**
**Residence**			
Rural	Ref		
Town	5.47	0.66—45.43	**0.115**
**Occupation**			
Unemployed	Ref		
Employed	4.68	1.35—16.25	**0.015**
**Education level**			
Primary/none	Ref		
Secondary or higher	2.27	0.69—7.48	**0.179**
**Religion**			
Protestant	Ref		
Catholic	1.66	0.39 -7.07	0.496
Other	2.57	0.42—15.92	0.3
**Shelter**			
Yes	2.6	0.30—22.46	0.385
No	Ref		
**Household head**			
Yes	0.83	0.16—4.38	0.829
No	Ref		
**Household member number**			
4–7	Ref		
> 7	0.34	0.10—1.24	0.102
**Monthly allowance**			
< 10 $	Ref		
> 10 $	7.65	0.92—62.92	0.059

Markers of sex and occupation were determinants associated with quality of life on bivariate analysis *(p* = 0.01), see Table 4

Muscle pain and experience of stress were not determinants associated with quality of life on bivariate analysis (*p* = 0.619), see Table 5.

**Table 5:** Bivariate analysis for medical and community determinants

Variables	Unadjusted OR	95% Cl	*P*-value
**Medical determinants**			
**Muscle pain**			
Yes	1.4	0.37—5.27	0.619
No	Ref		
**Chest Pain**			
Yes	0.44	0.05—3.87	0.461
No	Ref		
**Fatigue**			
Yes	0.79	0.19—3.21	0.73
No	Ref		
**Sleep problem**			
Yes	0.73	0.14—3.81	0.712
No	Ref		
**Community-based Care determinants**			
**Stigma**			
Yes	0.69	0.20—2.04	0.563
No	Ref		
**Experienced Stress**			
Yes	1.04	0.20—5.57	0.96
No	Ref		
**Social support**			
Yes	0.32	0.08—1.26	0.103
No	Ref		

On multiple analysis, sex and place of residence significantly influenced quality of life of caregivers of adolescents and young adults Ebola survivors, male caregivers were 3.17 times more likely to record good quality of life than female caregivers *(p* = 0.02); OR:(95%CI), 3.17: (1.2 – 8.36), caregivers who lived in town were less likely to have good quality of life compared to those in rural (*p* = 0.01); OR:0.25: (0.09 – 0.72) see Table 6.

**Table 6:** Multivariate analysis for socio-demographic, medical and community determinants

Variables	OR	95% CI	*P*-value
**Sex**			
Male	3.17	1.20 – 8.36	**0.02**
Female	Ref		
**Marital status**			
Married	0.95	0.24—3.73	0.941
Single	Ref		
**Residence**			
Town	0.25	0.09 – 0.72	**0.01**
Rural	Ref		
**Occupation**			
Unemployed	0.75	0.25 – 2.19	0.594
Employed	Ref		
**Education level**			
Secondary or higher	2.28	0.68 – 7.71	0.184
Primary/ none	Ref		
**Household size**			
> 7	0.26	0.06 – 1.13	0.072
4 – 7	Ref		
**Monthly allowance**			
> 10 $	4.45	1.65 – 30.35	0.127
< 10 $	Ref		
**Social support**			
Yes	0.37	0.11 – 1.225	0.109
No	Ref		

## Discussion

### Quality of life of caregivers of adolescent and young adult Ebola survivors

The purpose of this study was to investigate determinants of quality of life among caregivers of adolescent and young adult Ebola survivors in the Democratic Republic of the Congo. This study reported a high prevalence of poor quality of life among caregivers, with three quarters having poor quality of life. This was extremely high compared to findings from a study in Uganda on QOL of caregivers of cancer patients, which reported less than half of the caregivers to have poor quality of life. This kind of divergence could be explained by the difference in the dynamics of the diseases, geographical regions and economic abilities of the two nations, given the long history of political instability in the DRC. Additionally, the effect of Ebola virus on its survivors and caregivers is profound. Given its severity, caregivers suffer psychological distress as a result of fear of infection, or loss of a loved one [12]. Furthermore, the traumatic experiences and possibility of death, profoundly affect both survivors’ and caregivers’ psychological wellbeing which consequently impact their quality of life [13].

Sex of the caregivers was a significant determinant of their QOL in this study. Men were highly likely to have good quality of life than women. The gender discrepancy in viewpoints of illness perception sequence may contribute to female caregivers’ lower level of quality of life than men.

This finding is consistent with findings from studies by Sun Young et.al. and Shao-Yin et al., which also reported men to have higher QOL than women caregivers [14, 15]. Another study however found a negative association between male caregivers and quality of life [16]. A study from Indonesia did not find gender to have significant influence on quality of life of caregivers [17].

It was also established from the study that place of residence had significant impact on QOL of caregivers of Ebola survivors. Inherently, people’s interaction with their residential environments plays a vital role in their health. Their interpersonal and social associations in these environments influences their quality of life [18]. Urban residence in our study was negatively associated with quality of life of caregivers. This could be credited to high rates of capitalism, poverty, lack of affordable housing, pollution, and slum creation among others in urban settings [19]. A limited number of studies have examined the relationship between place of residence and quality of life of caregivers. However, the study revealed that place of residence significantly influences QOL of individuals [18].

The study found no significant association between QOL and marital status, occupation, social support and monthly allowance of caregivers. However, there is a substantial number of studies that have reported association between education, monthly income, social support and QOL of caregivers [14, 20, 21]. Similar to our findings, a recent study by Kizza, found no significant association between social support and QOL [20].

The study found no significant association between QOL and marital status, occupation, social support and monthly allowance of caregivers. However, there is a substantial number of studies that have reported association between education, monthly income, social support and QOL of caregivers [14, 20, 21]. Similar to the study findings, a recent study by Kizza, found no significant association between social support and QOL [20].

## Conclusions

Three quarter of caregivers of adolescents and young adult Ebola virus survivors have poor quality of life and being women caregiver and living in town are associated determinants with poor quality of life among caregivers of adolescent and young adult Ebola survivors in Democratic republic of the Congo.

## Data Availability

All data generated and analyse during current study is available in given article and also available from corresponding author upon reasonable request.

## Abbreviations

QOL: Quality of life
WHOQOL-BREF: Word Health Organization Quality of Life abbreviated questionnaire
